# Selection into medical school: from tools to domains

**DOI:** 10.1186/s12909-016-0779-x

**Published:** 2016-10-03

**Authors:** Tom M. Wilkinson, Tim J. Wilkinson

**Affiliations:** 1 Hawkes Bay Hospital, Hastings, New Zealand; 2 Medical Education Unit, University of Otago, P.O. Box 4345, Christchurch, 8140 New Zealand

**Keywords:** Selection, Blueprinting

## Abstract

**Background:**

Most research into the validity of admissions tools focuses on the isolated correlations of individual tools with later outcomes. Instead, looking at how domains of attributes, rather than tools, predict later success is likely to be more generalizable. We aim to produce a blueprint for an admissions scheme that is broadly relevant across institutions.

**Methods:**

We broke down all measures used for admissions at one medical school into the smallest possible component scores. We grouped these into domains on the basis of a multicollinearity analysis, and conducted a regression analysis to determine the independent validity of each domain to predict outcomes of interest.

**Results:**

We identified four broad domains: logical reasoning and problem solving, understanding people, communication skills, and biomedical science. Each was independently and significantly associated with performance in final medical school examinations.

**Conclusions:**

We identified two potential errors in the design of admissions schema that can undermine their validity: focusing on tools rather than outcomes, and including a wide range of measures without objectively evaluating the independent contribution of each. Both could be avoided by following a process of programmatic assessment for selection.

## Background

The selection of medical students is controversial and areas of consensus are rare [1–3]. A number of selection tools are used, including prior academic achievement, aptitude tests, interviews, personal statements, and personality tests [1–4]. Medical schools differ widely in their choice of tools and the relative importance of each. Furthermore, there is heterogeneity within each tool: for example, the interview used by one medical school may lead to different conclusions to the interview of another school [3].

A recent systematic review of research into medical student selection noted there has been very little research exploring construct validity issues i.e. what is being measured [1]. Instead, much has focused either on reliability, or on evaluating the validity of a particular tool in isolation, in an attempt to determine how well it predicts later performance - later performance being generally measured by examinations [2, 3]. The usefulness of such research can be limited: conclusions about the validity of a tool at one institution may not apply to the equivalent tool used at another institution, and it is difficult to apply any findings practically with any objectivity. Bridging the gap from such research to an evidence-based overall admissions scheme is difficult. An Australian study recognised this problem and produced a proposed scheme, but even here there was no analysis linking the scheme to later outcomes [4].

Recent calls have been made for a movement to programmatic assessment for selection [2, 3]. This would entail a focus on an overall admissions scheme, with explicit identification of the domains being selected for, justification of each, and linkage of those domains to selection tools.

Data on the isolated correlation of a selection tool with later examination performance is of limited value in this process, as it fails to account for what that tool is measuring, and how this may relate to other components of the same admissions scheme. For example, the multiple mini-interview (MMI) [5] in one school may measure something different in another school, despite both being identified as “MMI”. Furthermore, it has been demonstrated that such analyses can lead to skewed conclusions [6]. Another example is the Undergraduate Medical Admissions Test (UMAT), which has three components. While the predictive validity of each of the three components has been studied in a research setting, in practice there is heterogeneity in its use [7] and often the aggregate score is used to make selection decisions [8].

We therefore draw on the issues and gaps raised by a recent systematic review [1] and apply the conceptual framework of programmatic assessment.

This study aims to produce a test of concept and an evidence-based blueprint for programmatic assessment in selection. The context of this study, at Otago Medical School (New Zealand), offers an important advantage: most students are selected following a common first year at university. This enables a pool of students to be studied who have results from the same set of measures. Thus, while the context is limited to one university, the principles we are applying, as a test of concept, might be broadly applicable.

We aim to divide our current selection tools into domain-specific components, evaluate the unique contribution of each component to the overall predictive validity of the admissions scheme, and use this to produce a blueprint listing those domains with predictive validity and the relative importance of each.

More broadly, we aim to make this blueprint applicable to a broad range of admissions schema, and to test the potential of a method through which other institutions can produce blueprints of their own.

## Methods

Under the main admissions pathway for entrance into the medical programme at Otago University (New Zealand) students complete an open-entry first year course before applying for entry into second year medicine. Applicants complete seven courses during this year, mainly in biomedical science. They are also required to sit the UMAT, an aptitude test used by most Australasian undergraduate medical schools, comprising three sections. Therefore ten separate admissions measures (and potential predictors) are available for each student. Each generates a possible score on a scale of 0–100. Details of these measures are presented in Table 1.

**Table 1:** Measures of Admission (2003–2006)

*Compulsory first year courses*	
• Anatomy and Physiology	
• Biochemistry	
• Cell and Molecular Biology	
• Chemistry	
• Physics	
• Epidemiology and Public Health	
• Communication Skills	
*UMAT*	
• Section One: Logical Reasoning and Problem Solving	
• Section Two: Understanding People	
• Section Three: Non-Verbal Reasoning	

For the purposes of analysis we required cohorts of students who completed the same admissions process, and who could be followed 5 years later to determine outcomes. The ten admission measures changed in both 2003 and 2007. Therefore, our study cohort consisted of all students who were successfully admitted to the medical course after completing the introductory first year between 2003 and 2006 inclusive. As the admissions process guided our cohort selection, no power calculations were performed.

The medical course is six years long (including the introductory first year), with the final common examinations occurring at the end of year five. These examinations form the outcome of interest in our study, and comprise an OSCE and three written exams (two comprising multiple choice questions and one comprising short answer questions). The predictive validity of these examinations has been reported previously, with a combined score (60 % OSCE, 40 % written) shown to be a good predictor of later performance [9]. This combined score was therefore used as our primary outcome, however secondary analyses were also performed for OSCE score alone, and for written exam score alone.

Our statistical analysis occurred in two parts.

Firstly, we constructed correlation matrices, between all ten measures of admission, and all three possible outcomes, using the Pearson correlation coefficient. If the correlation between a measure of admission and our primary outcome (aggregate fifth year exam score) was not significant at *p* < 0.05, then we considered that measure of admission to be of limited benefit in an admissions model, and removed it from further analysis.

We used these same correlation matrices to assess for multicollinearity between the ten admission measures. Any measures with a correlation *r* > 0.4 were considered to be at potential risk of multicollinearity. When this occurred the measures concerned were grouped into a thematic domain.

Secondly, we used simple linear regression to assess the relative importance of each of the remaining admission measures. All such measures were regressed against each of the three possible outcomes, producing three separate regression models. For each model, the relative importance of each thematic domain was calculated as the sum of the standardised *β*-coefficients of its component measures. Statistical significance was calculated using partial F-tests.

## Results

We obtained data for all 507 students who completed all ten admissions measures, subsequently gained entry to medical school, and completed the fifth year medical school examinations.

Correlation matrices are presented in Tables 2 and 3.

**Table 2:** Correlation between measures of admission and outcomes (Pearson’s r)

	Year 5 written exam	Year 5 OSCE	Year 5 aggregate mark
UMAT section 1 (“Logical reasoning and problem solving”)	0.23**	0.16**	0.22**
UMAT section 2 (“Understanding people”)	0.19**	0.22**	0.24**
UMAT section 3 (“Non-verbal reasoning”)	0.01	−0.02	−0.01
Human biology course	0.34**	0.23**	0.31**
Biochemistry course	0.32**	0.24**	0.31**
Cellular biology course	0.30**	0.18**	0.26**
Chemistry course	0.24**	0.00	0.11**
Physics course	0.22**	0.05	0.13**
Epidemiology and public health course	0.34**	0.20**	0.29**
Communication skills course	0.17**	0.22**	0.23**

*p < 0.05, **p < 0.01

**Table 3:**
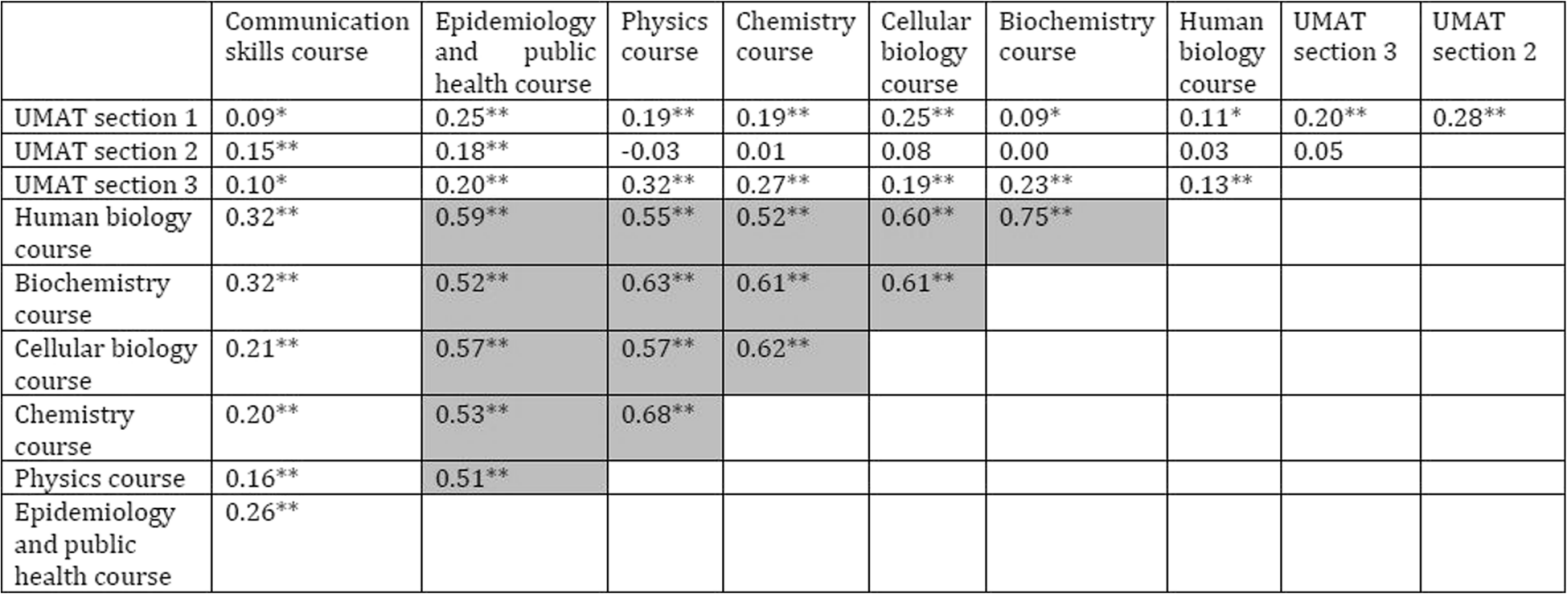
Correlation matrix for measures of admission (Pearson’s r)

*p < 0.05, **p < 0.01, cells highlighted where *r* > 0.4, suggesting multicollinearity

The admissions measure, UMAT section 3 (“non-verbal reasoning”) was not significantly correlated with our primary outcome, and was therefore removed from further analysis.

Six admissions measures were significantly correlated with each other at *r* > 0.4, and were therefore grouped together into a common domain (human biology course, biochemistry course, cellular biology course, chemistry course, physics course, epidemiology and public health course). The reliability of this biomedical science domain, as assessed by Cronbach’s alpha, was 0.92. No further correlations of *r* > 0.4 were observed between the admissions measures. Therefore, the three remaining measures (UMAT section 1, UMAT section 2, and communications skills course) were assessed independently.

Regression analysis is presented in Table 4. All four tested domains were independently and significantly (*p* < 0.01) correlated with our primary outcome (year 5 aggregate mark).

**Table 4:** Regression analysis (standardised *β*-coefficients)

	Year 5 written exam	Year 5 OSCE	Year 5 aggregate mark
Domain 1			
UMAT section 1 (“Logical reasoning and problem solving”)	0.132**	0.195**	0.193**
Overall contribution	0.132**	0.195**	0.193**
Domain 2			
UMAT section 2 (“Understanding people”)	0.077**	0.159**	0.146**
Overall contribution	0.077**	0.159**	0.146**
Domain 3			
Human biology course	0.102	0.188**	0.175**
Biochemistry course	0.136*	0.086	0.121
Cellular biology course	0.160**	0.030	0.089
Chemistry course	−0.040	−0.218**	−0.166
Physics course	−0.060	−0.026	−0.047
Epidemiology and public health course	0.106	−0.054	0.009
Overall contribution	0.404**	0.006**	0.181**
Domain 4			
Communication skills course	0.056	0.177**	0.148**
Overall contribution	0.056	0.177*	0.148**
Overall model R^2^	0.206	0.183	0.213

* = *p* < 0.05, ** = *p* < 0.01, significance calculated from partial F-test

## Discussion

The results of this study provide information that could be used for a blueprint for medical admissions schemes and has some features that are consistent with principles of programmatic assessment [10], particularly where decisions are made by attribute than by tool.

The multicollinearity analysis grouped our admissions measures into four broad domains: Logical reasoning and problem solving, understanding people, communication skills, and a fourth broad domain that encapsulates biomedical science (human biology, cellular biology, biochemistry, chemistry, physics, and epidemiology). Each domain was independently and significantly associated with our primary outcome of overall performance in final medical school examinations. Furthermore, each domain was given a similar weighting in our regression model.

The argument for validity is based on consistency of associations between similar constructs and the associations with elements of medical practice needed by doctors [11]. Construct validity is supported by noting the differential correlations of domains with outcomes – particularly the OSCE having stronger correlations with communication skills and understanding people, and weaker correlations with biomedical science. That is, the associations between admission measures and final performance are in ways that might be expected. We also suggest that these four domains have a certain face validity, as they resemble some of the attributes most would like to see in future doctors [12]. We acknowledge however that important attributes, such as professionalism, empathy etc., are not included within these domains.

We suggest that using these four domains (with an equal weighting on each) could form a validated blueprint on which to map assessment tools for medical school admissions.

We do make note of possible negative associations seen in our modeling, for courses in chemistry, physics, and epidemiology. In the presence of multicollinearity these may well be spurious – however they do merit further investigation.

This study suggests that UMAT section 1 and UMAT section 2 are both valuable selection tools – each being significantly correlated with important medical school outcomes. However, UMAT section 3 does not appear to be useful. Previous studies investigating the validity of the UMAT have found similar differences in the validity of each section [13, 14], as have studies investigating the GAMSAT and BMAT [15, 16].

Within this cohort, communication skills were assessed by means of a university course with written assessments. This performed well as a predictor in our model, with a standardised β of 0.148. In comparison, a similar analysis performed by McMaster medical school reported standardised β values of 0.12 and 0.21 for the associated between their MMI and the Medical Council of Canada Qualifying Examination, part 1 and 2 respectively [17]. This course was also found to be largely independent of other university courses included in our analysis.

Our findings highlight possible limitations to constructing an admissions scheme around tools, rather than domains.

Aptitude tests, such as UMAT, contain a mixture of information. The introduction of such a test should include consideration of what information is wanted – and what isn’t. Our analysis suggests that only the first two UMAT sections (which map to validated domains) are useful. Grouping these two sections with UMAT section three, as many schools do in practice [8], may undermine their utility.

Conversely, there may be multiple valid ways of assessing a given domain. It may be easier to assess communication skills by way of a written exam than by way of an interview – and this appeared to perform well in our cohort.

Although this study was conducted within the context of admissions at one medical school, the findings have broader relevance. An advance in medical admissions has been the introduction of the multiple-mini interview (MMI), which has been shown to have promising validity and is now in use as part of many selection processes [17–19]. The programmatic approach suggested by our findings might explain why the MMI is useful. As a multiple-station assessment conducted by a medical school, the MMI is very conducive to being blueprinted against a predefined set of desirable attributes. The Canadian Dental Association structured interview (used in admissions for Canadian Dental Schools) has been shown to have particularly good validity, and it has been suggested that this reflects a strong underlying blueprint [2, 20]. However, existing frameworks for MMI’s are variable, with a lack of evidence for each included domain [3]. Thus, even the MMI, as a tool, can be confused with a domain. Instead, we suggest the domains of interest should be mapped to a MMI, alongside other tools that also assess domains of interest.

Our analysis has shown that some domains are represented by a single tool. Taking a focus on domains rather than tools need not imply that a tool cannot represent a domain. Instead we are suggesting that thinking first about domains and then judiciously choosing the right tool(s) is preferable to thinking first about tools.

In taking this view, we also do not mean to imply that what current medical schools are doing is wrong. In fact, the result of our final blueprint is not dissimilar to the admissions schemes used by many medical schools - the difference is in the process used to arrive at the blueprint. Even if the final outcome is the same, using a more robust process to reach that outcome is an improvement.

We present the findings of this study as a proof of concept, but it does have important limitations. Firstly, all presented results were derived from optimized models that have not been evaluated on a second cohort. All presented figures should be interpreted with caution. In particular, values for overall model R^2^ may be larger than would be obtained if this model were applied to a new cohort. In contrast, restriction of range might have falsely reduced the correlations seen because students who get into medical school have results that are nearer the top end of any range of scores. However, this does not undermine the validity of our conclusions regarding principles underpinning the design of admissions schema, nor does it undermine the relative weightings that we have derived.

Secondly, there may be other domains that have independent predictive importance that our existing admissions tools fail to accurately capture. Such domains might include professionalism, probity, empathy, teamwork etc. This could be a promising area for future research.

Finally, such a scheme does not address social accountability or ways to increase the diversity of the medical student pool – each of which require separate approaches other than the simple measurement of domains.

## Conclusion

Our analysis has demonstrated a method for taking an approach to admissions that is focused on domains or attributes, rather than tools. As a test of concept we suggest that this may be of use for medical schools in evaluating their own admissions schema.
